# Causes of global extinctions in the history of life: facts and hypotheses

**DOI:** 10.18699/VJ20.633

**Published:** 2020-07

**Authors:** T.M. Khlebodarova, V.A. Likhoshvai

**Affiliations:** Institute of Cytology and Genetics of Siberian Branch of the Russian Academy of Sciences, Novosibirsk, Russia; Institute of Cytology and Genetics of Siberian Branch of the Russian Academy of Sciences, Novosibirsk, Russia

**Keywords:** Earth’s fossil record, evolution of global ecosystems, mass extinctions, dynamic systems, complex dynamics, periodicity, modeling, палеонтологическая летопись Земли, эволюция глобальных экосистем, массовые вымирания, динамические системы, сложная динамика, периодичность, моделирование

## Abstract

Paleontologists define global extinctions on Earth as a loss of about three-quarters of plant and animal species over a relatively short period of time. At least five global extinctions are documented in the Phanerozoic fossil record (~500-million-year period): ~65, 200, 260, 380, and 440 million years ago. In addition, there is evidence of global extinctions in earlier periods of life on Earth – during the Late Cambrian (~500 million years ago) and Ediacaran periods (more than 540 million years ago). There is still no common opinion on the causes of their occurrence. The current study is a systematized review of the data on recorded extinctions of complex life forms on Earth from the moment of their occurrence during the Ediacaran period to the modern period. The review discusses possible causes for mass extinctions in the light of the influence of abiogenic factors, planetary or astronomical, and the consequences of their actions. We evaluate the pros and cons of the hypothesis on the presence of periodicity in the extinction of Phanerozoic marine biota. Strong evidence that allows us to hypothesize that additional mechanisms associated with various internal biotic factors are responsible for the emergence of extinctions in the evolution of complex life forms is discussed. Developing the idea of the internal causes of periodicity and discontinuity in evolution, we propose our own original hypothesis, according to which the bistability phenomenon underlies the complex dynamics of the biota development, which is manifested in the form of global extinctions. The bistability phenomenon arises only in ecosystems with predominant sexual reproduction. Our hypothesis suggests that even in the absence of global abiotic catastrophes, extinctions of biota would occur anyway. However, our hypothesis does not exclude the possibility that in different periods of the Earth’s history the biota was subjected to powerful external influences that had a significant impact on its further development, which is reflected in the Earth’s fossil record.

## Abstract (ru)

Палеонтологи характеризуют глобальные вымирания на Земле как потерю ~3/4 существую- щего биоразнообразия на большей части земного шара за относительно короткий геологический про- межуток времени. В палеонтологической летописи Земли, описывающей период фанерозоя (~500 млн лет), документировано как минимум пять таких глобальных вымираний: ~65, 200, 260, 380 и 440 млн лет назад. Существуют данные о возможности глобальных вымираний в более отдаленные периоды жизни на Земле – в позднем кембрии (~500 млн лет назад) и эдиакарии (более 540 млн лет назад). Общего мнения о причинах их возникновения до сих пор не сформировано. В настоящем обзоре систематизированы до- кументированные факты глобальных вымираний сложных форм жизни на Земле с момента их возникно- вения в эдиакарии и до современного периода. Рассматриваются возможные причины их возникновения с точки зрения воздействия абиогенных факторов, планетарных или астрономических, и последствий их действия. Анализируются данные «за» и «против» гипотезы периодичности массовых вымираний био- разнообразия морской биоты в фанерозойский период. Обсуждаются факты, позволяющие высказывать гипотезы о наличии дополнительных механизмов возникновения кризисов в эволюции сложных форм жизни на Земле, связанных с различными внутренними биотическими факторами. Развивая тему внутрен- них причин периодичности и прерывистости эволюционного процесса, мы высказываем собственную, оригинальную гипотезу, согласно которой глобальные вымирания являются отражением сложной динамики изменения уровня биоразнообразия на Земле и следствием феномена бистабильности. Этот фено- мен возникает только в экосистеме, бóльшая часть организмов которой размножается половым путем. Данная гипотеза говорит о том, что, если бы даже не было никаких глобальных катастроф абиотическо- го характера, кризисы в развитии биоты возникали бы все равно. Однако гипотеза не исключает, что в определенные моменты времени биота Земли подвергалась мощным внешним воздействиям, оказавшим существенное влияние на ее дальнейшее развитие, что нашло отражение в конкретных палеонтологиче- ских данных.

## Introduction

Global extinctions on Earth are defined by paleontologists as a loss of about three-quarters of the existing biodiversity in a relatively short interval of geologic time. At least five global extinctions are documented in the Phanerozoic fossil record (~500 million years). These are the Cretaceous-Paleogene extinction event (~65 million years ago), the Triassic-Jurassic extinction event (~200 million years ago), extinction near the Permian-Triassic boundary (~260 million years ago), the late Devonian extinction (~380 million years ago), and extinction near the Ordovician-Silurian boundary (~440 million years ago). These five extinction events were first described as “Big Five” extinctions based on the analysis of more than 36 thousand kinds of marine invertebrate fossils, which were catalogued in the D.M. Raup and J.J. Sepkoski’s database (Raup, Sepkoski, 1982). Some researchers argue that a sixth mass extinction is currently underway on our planet. This opinion is based on the estimates of species extinction rates in the current period, which were found to be comparable to those during global extinctions estimated on the basis of paleontological data (Barnosky et al., 2011; Ceballos et al., 2015).

In the last decade, intensive analysis of fossil material has revealed new examples of mass extinctions of complex life forms on Earth. There is evidence that during the early periods of life on Earth – in the Late Cambrian (~500 million years ago) and during the Ediacaran period (> 540 million years ago) (Gill et al., 2011; Darroch et al., 2015), extinctions were global. Extinction during the Ediacaran period is considered to be the first mass extinction of complex life forms on Earth (Darroch et al., 2015). Let us consider the facts and hypotheses concerning the causes of global extinctions.

## Mass extinctions as a result of global disasters of an abiotic nature

A number of abiogenic factors has been described that could potentially cause most of the big extinctions detected in the Earth’s fossil record. This does not apply to the biodiversity loss during the late Ediacaran period (Xiao, Laflamme, 2009; Buatois et al., 2014; Darroch et al., 2015), the late Cambrian period (Gill et al., 2011), and the modern period (Barnosky et al., 2011; Ceballos et al., 2015).

The most well-known abiogenic factors that have been associated with the environmental disasters are: the struck of a massive asteroid ~65 million years ago (Alvarez et al., 1980, 1981; Schulte et al., 2010; Kaiho, Oshima, 2017), volcanic activity and global warming ~200 million years ago (Marzoli et al., 1999; Whiteside et al., 2010; Blackburn et al., 2013; Thibodeau et al., 2016; Miller et al., 2017; Percival et al., 2017; Heimdal et al., 2018), trappean eruptions ~260 million years ago (Huey, Ward, 2005; Wignall et al., 2009; Rampino et al., 2017), as well as the major Gondwanan glaciation and climate cooling ~440 million years ago (Sutcliffe et al., 2000; Sheehan, 2001; Finnegan et al., 2011, 2012; Sheets et al., 2016). These phenomena and their consequences associated with climate change allow us to explain, at least to a certain extent, the extinction near the Cretaceous-Paleogene boundary (Alvarez et al., 1980, 1981; Schulte et al., 2010; Kaiho et al., 2016), the Triassic-Jurassic extinction event (Marzoli et al., 1999; Whiteside et al., 2010; Blackburn et al., 2013; Percival et al., 2017), the Late Permian extinction (Wignall et al., 2009), and the extinction near the Ordovician-Silurian boundary (Sutcliffe et al., 2000; Sheehan, 2001; Finnegan et al., 2011, 2012; Sheets et al., 2016).

However, it should be noted that the described external influences during these periods are quite diverse and there is still no single opinion on the causes of known extinctions, especially regarding the Late Devonian extinction ~380 million years ago.

Therefore, analysis of another dataset demonstrates the link between the extinction near the Cretaceous-Paleogene boundary ~65 million years ago and the sea-level changes caused by movements of the tectonic plates (Peters, 2008) or volcanic activity (Archibald et al., 2010; Courtillot, Fluteau, 2010; Keller et al., 2010; Schoene et al., 2015, 2019).

Some researchers explain the Triassic-Jurassic extinction event ~200 million years ago by significant climate warming as a result of abnormally high concentrations of atmospheric carbon dioxide of magmatic origin (McElwain et al., 1999; Beerling, 2002; Schaller et al., 2011), which could be accompanied by storms, lightning strikes and, as a result, fires. The latter could directly cause the global extinction of the terrestrial biota (Petersen, Lindström, 2012). Some authors deny the link between the global biodiversity loss and changes in atmospheric carbon dioxide concentration during that period (Tanner et al., 2001). Other scientists attribute mass extinction to the emission of large volumes of volcanic sulphurous gas (Bacon et al., 2013) or to frequent warming and cooling of the climate caused by volcanic emissions of large volumes of sulphurous gas followed by carbon dioxide emission (Guex et al., 2016). Recent studies confirm the great impact of volcanic activity on the climate change at the end of the Triassic period and provide evidence that toxic effect of volcanic emissions can be associated with mercury – the most genotoxic element on Earth (Percival et al., 2017; Lindström et al., 2019).

Biodiversity loss during the Late Permian ~260 million years ago, when more than 90 % of marine invertebrates became extinct, has been explained by various reasons: low oxygen concentration in the surface layer of the ocean (Knoll et al., 1996; Wignall et al., 2009; Shen et al., 2011; Zhang et al., 2018a), including in combination with warm climate which is harmful to shallow-water organisms (Song et al., 2013); ocean acidification associated with carbon dioxide release into the atmosphere and the accompanying rapid global warming and acid rain (Clarkson et al., 2015; Sun et al., 2018); climate cooling, combined with aridity, hypoxia, and acid rain (Zhu et al., 2019). Mathematical modeling of the Late Permian climate supports the hypothesis that reduced biodiversity during that period could be due to hypoxia and ocean warming (Penn et al., 2018). Recently, additional data in favor of the volcanic hypothesis of the biotic crisis in the Late Permian period have been obtained (Burgess et al., 2017; Shen et al., 2019).

Biodiversity loss near the Ordovician-Silurian boundary ~440 million years ago, when ~85 % of marine organisms became extinct, has been traditionally associated with the global cooling of the tropical ocean (Sutcliffe et al., 2000; Sheehan, 2001; Finnegan et al., 2011, 2012), which was accompanied by a drop of the sea level and the loss of shallow habitats (Finnegan et al., 2012).

According to some researchers, such cooling was triggered by a significant increase in cosmic dust in the inner space of the solar system due to the decay of the L-chondrite parent body in the asteroid belt ~466 million years ago (Schmitz et al., 2019), while others deny the connection between the asteroid destruction and the level of biodiversity (Lindskog et al., 2017).

Some researcher believe that scenario of the Ordovician- Silurian extinction was more complicated, included three ice ages and the cause of the initial extinction was not the sea cooling, but the ice melt from glaciers due to the presence of a large ice cover and a relatively warm ocean during that period causing sea level to rise (Ghienne et al., 2014). The cause of the second extinction has been considered to be the decreased oxygen concentration in water that occurred when the sea level was high before the glaciation peak in the Late Ordovician period (Bartlett et al., 2018). Nowadays, volcanic activity is considered to be the cause of the second extinction (Gong et al., 2017; Rasmussen et al., 2019; Smolarek-Lach et al., 2019).

There are many different hypotheses about the cause of the Late Devonian extinction ~380 million years ago (Sallan, Coates, 2010), which mainly affected the marine biota, especially in shallow water (Ma et al., 2016). Some researchers associate it with climate cooling (Huang et al., 2018; Wang et al., 2018), which was provoked by the burial of a large amount of organic carbon with a subsequent decrease in atmospheric carbon dioxide concentration (Huang et al., 2018), and was accompanied by the sea-level decrease (Wang et al., 2018). Others attribute the Devonian extinction to global warming caused by a massive release of methane gas into the atmosphere, which could be caused by volcanic activity (Gharaie et al., 2004, 2007). And others link it to the frequent climate change from warming to cooling (Chen et al., 2005), which was accompanied by sea level fluctuations (Joachimski, Buggisch, 1993) and was provoked by various processes, including the burial of a large amount of organic carbon and the dissociation of gas hydrates (Chen et al., 2002). Devonian extinction has also been associated with the spread of fires, the cause of which is considered to be the high concentration of atmospheric oxygen together with dry climate (Kaiho et al., 2013), trap eruptions (Ricci et al., 2013), asteroid fall (Claeys et al., 1992), etc. It is overall recognized that causes of Devonian extinction are still not clear (Percival et al., 2018).

It is also worth noting the potential uniqueness of biotic crises during the late Devonian period and near the end of the Triassic period, which were associated not with an increased extinction rate, but with a decrease in the rate of speciation (Bambach et al., 2004; Lamsdell, Selden, 2017).

As for the remaining documented extinctions: during late Cambrian period ~499 million years ago (Gill et al., 2011), near the end of the Ediacaran period > 540 million years ago (Xiao, Laflamme, 2009; Buatois et al., 2014; Darroch et al., 2015; Zhang et al., 2018b), as well as the loss of biodiversity observed in the modern period (Barnosky et al., 2011; Ceballos et al., 2015), they have not been associated with global catastrophes of an abiotic nature.

Recently, the lack of oxygen in water is more and more often considered one of the main causes of global extinctions of biota, including during the Ediacaran period (Zhang et al., 2018b), during the Late Cambrian period (Gill et al., 2011), near the Ordovician-Silurian boundary (Bartlett et al., 2018), during the late Devonian period (Bond, Wignall, 2008; Liu et al., 2016), at the end of the Permian period (Brennecka et al., 2011; Shen et al., 2011; Lau et al., 2016; Zhang et al., 2018a), and during the early Jurassic period (Them et al., 2018). However, if during the late Permian period the lack of dissolved oxygen is believed to be a consequence of a global warming (Zhang et al., 2018a), and during the late Ordovician period – a consequence of a climate cooling (Bartlett et al., 2018), what could cause it during other periods of mass extinctions is not yet clear. Moreover, there is evidence (Darroch et al., 2015) that contradicts the assertion (Zhang et al., 2018b) of oxygen deficiency in the late Ediacaran ocean.

## Periodicity in the history of global extinctions

**Fig. 1.**
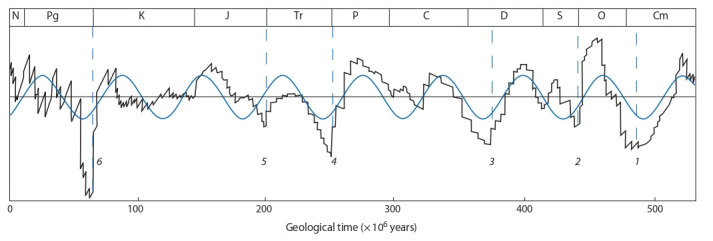
Diversity dynamics of the Phanerozoic marine biota. The main geological periods are indicated above, according to (Raup, Sepkoski, 1982), the vertical dashed line shows the times of six global extinctions of biota on Earth: 1 – during the Late Cambrian; 2 – near the Ordovician-Silurian boundary; 3 – during the late Devonian period; 4 – near the Permian-Triassic boundary; 5 – near the Triassic-Jurassic boundary; 6 – near the Cretaceous-Paleogene boundary. Blue curve is a sine wave, black curve is adapted from (Rohde, Muller, 2005; Fig. 1, c).

It is important to note that episodes of mass extinctions on the Earth are strongly believed to be cyclical, which was first noted when creating the first comprehensive database on the fossil record of marine families during the Phanerozoic period (Raup, Sepkoski, 1984, 1986; Sepkoski, 1989). Over a time span of 250 million years, eight largest extinction-intensity peaks with a periodic fluctuation in marine biodiversity of ~26–27 million years have been detected. Since then, data from the Sepkoski’s dataset (Sepkoski, 2002) have been intensively analyzed using various methods; some authors report the presence of a slightly pronounced periodicity of extinctions of ~27 million years (Lieberman, Melott, 2007), whereas data obtained by other researchers indicate a strict periodicity of ~62–63 million years (Rohde, Muller, 2005; Lieberman, Melott, 2007), which appeared over an interval of 500 million years (Rohde, Muller, 2005) (Fig. 1).

Similar studies were conducted using alternative databases: Paleobiology Database (PBDB) of marine invertebrate fossils (Alroy, 2008; Melott, 2008; Lieberman, Melott, 2012; Roberts, Mannion, 2019) and Fossil Record 2 databases for marine and terrestrial fossils (Benton, 1995). Data were obtained both in favor of the presence of periodicity (Melott, 2008; Lieberman, Melott, 2012; Roberts, Mannion, 2019) and against strict cyclicality (Benton, 1995; Alroy, 2008).

In the study (Benton, 1995) seven peaks of mass extinctions of marine families were identified within the past 250 million years with a time interval between them varying from 20 to 60 million years. As for the PBDB material, the results of the study (Alroy, 2008) did not reveal any evidence in favor of periodic extinctions. However, other results confirmed the existence of a fairly strict periodicity of ~62–63 million years in the occurrence of major extinction events in the Phanerozoic (Melott, 2008; Lieberman, Melott, 2012), which was also shown in the analysis of the Sepkoski’s dataset (Rohde, Muller, 2005; Lieberman, Melott, 2007, 2012). Recent studies of the Paleobiology Database (Roberts, Mannion, 2019) confirm the extinction periodicity of ~27 million years, but limit them to the last 200 million years. The certainty and significance of the cyclical nature of extinctions with periods of ~27 and ~62 million years in the last 465 million years has been demonstrated in other studies (Melott, Bambach, 2014, 2017).

It is necessary to add that one more cycle of marine biodiversity change with a period of 140 ± 15 million years was found in the analyses based on the Sepkoski’s dataset (Rohde, Muller, 2005), but cyclicality of global extinctions in Phanerozoic with ~62–63 million years period was more strict.

Hense, based on various databases, researchers have reported at least three cycles of mass extinctions with periods of 26–30, 62–63, and ~140 million years during the Phanerozoic eon (Raup, Sepkoski, 1984, 1986; Sepkoski, 1989; Rohde, Muller, 2005; Lieberman, Melott, 2007, 2012; Melott, 2008; Melott, Bambach, 2014, 2017; Roberts, Mannion, 2019). A cycle with a period of ~27 million years was most clearly manifested during the last 200 million years (Roberts, Mannion, 2019).

In this regard, the question arises – is there a connection between the observed periodicity in the diversity of terrestrial biota and those processes that are considered above to be causes of global extinctions? In other words, is there a periodic abiotic process that could underlie the observed periodicity in the diversity of marine or terrestrial biota or even Earth’s entire biota?

Here it is important to emphasize once again that extinctions described above are global, that is, they affect almost the entire Earth’s biota, which means that if observed periodicity was associated with abiotic factors, it could reflect only those processes that affect the entire planet and are cyclical. From this point of view, two types of processes that have similar characteristics can be distinguished. The first are “inside planetary” processes, that is, they are associated with dynamic processes involved in plate tectonic motion that lead to continental drift, volcanic activity, changes in sea level, etc. The second are associated with extra-planetary influences and are a reflection of processes associated with the dynamics of the planet itself being a space object interacting with other objects of the universe.

Let us consider the existing hypotheses on the relationship between the periodicity of global extinctions and global catastrophes, which could be caused by such cyclical processes.

## Frequency of extinctions as a reflection of planetary processes and the evolution of the Sun

Nowadays, there is a number of hypotheses regarding possible connection between the periodicity of extinctions on Earth and astronomical processes. For example, a model of large-scale fluctuations in the magnetic field of the Sun shows an impressive periodicity of 66 million years (Baker, Flood, 2015), which is very close to the periodicity of mass extinctions of ~62–63 million years identified by analyzing at least two databases of marine invertebrate fossils (Rohde, Muller, 2005; Lieberman, Melott, 2007, 2012). Other hypotheses have been proposed linking frequency of extinctions with fluctuations of extragalactic cosmic-ray intensity as a result of vertical oscillations of the solar system about the galactic plane (Medvedev, Melott, 2007); with the periodicity of the solar system passage through the plane of the Milky Way galaxy (Rampino et al., 1997, 2015; Lieberman, Melott, 2012); and with the periodicity of the passage of comets near Earth and the fall of asteroids, which can form different periodicities depending on the size of celestial body (Rampino, Stothers, 1984; Rampino et al., 1997).

However, in recent years, new findings indicate that periodicities associated with solar system oscillations about the galactic plane are statistically unreliable (Erlykin et al., 2017, 2018) and could not cause the periodicity of extinctions on Earth. And, although some researchers disagree, it is generally recognized that there is no direct evidence of astronomical reasons for the periodicity of biota extinctions on the planet Earth (Melott, Bambach, 2017).

As for the planetary processes, there is also a wide variety of opinions. Some researchers explain changes in the fossilized organisms by periodic changes in sea level (Peters, 2008; Tennant et al., 2016) or connect them with the dynamics of tectonic movement of continental plates and their fragmentation (Valentine, Moores, 1970; Zaffos et al., 2017). One of the assumptions regarding the fact that tectonic processes on Earth could cause periodicity of mass extinctions has been based on the data on the 60-million-year periodicity of seawater Sr87/ Sr86 ratio in marine sediments (Melott et al., 2012).

Other researchers detect a definite correlation between the biodiversity dynamics and the temperature regime on Earth (Mayhew et al., 2012) and consider periodic global climate changes to be the cause of extinctions. It can be noted here that glacial-interglacial cycles on Earth had a periodicity of ~135 million years (Veizer et al., 2000), which is statistically indistinguishable from the periodicity of 140 ± 15 million years, which was revealed based on the Sepkoski’s dataset (Rohde, Muller, 2005).

Of interest is the volcano crater dating over the past 260 million years, which demonstrates the cyclicity close to 26–27 million years (Rampino, Caldeira, 2015) characteristic of this particular period of time (Raup, Sepkoski, 1984, 1986; Sepkoski, 1989; Roberts, Mannion, 2019). However, in general, volcanic activity during the last 300 million years is characterized by weakly manifested cycles with a period of 15, 30, and 60 million years (Prokoph et al., 2004).

As for the rather strict ~62–63 million-years mass-extinction cycle identified by different researchers using different databases of marine invertebrate fossils (Rohde, Muller, 2005; Lieberman, Melott, 2007, 2012; Melott, 2008; Melott, Bambach, 2014, 2017), the existing data on 60-million-year periodicity associated with the dynamic processes involved in plate tectonic motion (Melott et al., 2012) and modeling data on the large-scale fluctuations of the solar magnetic field, both show periodicity of 66 million years (Baker, Flood, 2015), but do not allow strong connection with the periodicity of global extinctions on Earth.

Several times in the history of biological life on Earth have we detected serious external influences such as fall of asteroids and meteorites without subsequent extinction (Archibald et al., 2010), as well as extinctions without abiotic catastrophes, which leads us to an assumption that internal causes of a biotic nature could underlie mass extinctions of biota, which at different periods could coincide with global catastrophes or be provoked by them. We believe that these internal causes may be a reflection of a complex dynamic behavior of a living system, such as terrestrial or marine biota, or even the biota of the entire Earth.

## Mass extinctions and their periodicity as a reflection of internal properties of a global ecosystem

The idea that fossil biodiversity on Earth is a reflection of the internal laws of functioning of a global ecosystem, which is the Earth’s biota, has arisen more than once. Mass extinctions, which have been observed in the Earth’s fossil record over the past 500 million years and lead to intermittent and irregular evolutionary pace, represent just one aspect of the complex dynamic behavior of a global ecosystem. To explain the phenomenon of punctuated evolution, S.J. Gould and N. Eldredge have formulated the “theory of punctuated equilibrium” back in 1972 (Gould, Eldredge, 1977, 1993; Eldredge, Gould, 1997).

This theory is not strict. It is based on “empirical generalizations” of a number of facts that have long been noticed by paleontologists, which indicate that long periods of evolutionary stability, when species remain almost unchanged, alternate with short intervals of rapid qualitative change, which are characterized by “sudden” extinction of old species and subsequently replacement by new types. The authors of this theory and other researchers have found quite striking examples in the Earth’s fossil record confirming such pattern (Ovcharenko, 1969; Bambach, 1977; Gould, Eldredge, 1977, 1993; Williamson, 1981; Sepkoski, 1988; Jackson, Cheetham, 1999). Although the interpretation of some studies has been questioned (Van Bocxlaer et al., 2008), in general, presence of such pattern in the evolutionary process is not denied (Hunt, 2007; Mattila, Bokma, 2008; Rasskin-Gutman, Esteve-Altava, 2008).

Previously, the idea of internal biotic causes that determine the evolutionary dynamics was formulated as “self-organizing criticality” (Bak, Paczuski, 1995; Sneppen et al., 1995; Solé, Manrubia, 1996), which reflects interactions between different ecosystems and was used to explain mass extinctions and the hypothesis of punctuated evolution. It was assumed that these interactions, together with spontaneous mutations and genetic variations that are always present in populations, could lead to large evolutionary rearrangements called the “co-evolutionary avalanches”. Recently, the concept of “self-organizing criticality” has again attracted the attention of researchers (Nykter et al., 2008; Solé et al., 2010; Hesse, Gross, 2014; Valverde et al., 2015). However, already in the 1990s (Newman, 1997a, b) and later (Alroy, 2008), arguments against this concept have been expressed, which were based on the demonstration of the possibility of mass extinctions using simple models of species adaptation to existing conditions and nutrition resources without involvement of co-evolution and critical processes, both with and without influence of the abiotic factors (Roberts, Newman, 1996; Newman, 1997a, b).

There exist other ideas on the internal biotic causes of the biodiversity on Earth that relate the Phanerozoic biodiversity to the intensity of predation in marine communities (Huntley, Kowalewski, 2007) and suggest a certain role for predators in the formation of marine biota diversity, although no correlation between predators and preys were found in other studies (Madin et al., 2006). Other researchers, seeing a definite relationship between biodiversity and the age of the oceanic crust, connect the history of the seafloor with the biodiversity level via the availability of food resources (Cermeño et al., 2017).

In the existing models of the diversity dynamics of the Phanerozoic marine biota that has clear signs of punctuated evolution in its development, the periodicity of extinctions was not examined and was introduced into the models as a given (Markov, 2001a, b; Markov, Korotaev, 2007). However, discussing the modeling results, the authors noted that the causes of “staging” should be sought in the structure of developing communities (Markov, 2001a). A.V. Markov and A.V. Korotaev (2007) paid special attention to those life forms that have an increased adaptive capacity associated with sexual reproduction. In this regard, we should pay attention to the studies of A.M. Bush et al. (2016) who believe that diversification of marine predators starting from the Cretaceous- Cenozoic period (~200 million years ago) can be explained by the peculiarities of sexual reproduction during the directed transfer of sperm. However, given that internal fertilization has probably developed as early as in the late Neoproterozoic Era (> 500 million years), such delayed diversification requires an explanation (Novack-Gottshall, 2016).

A number of theoretical studies has connected discontinuity and staging in the Earth’s fossil record with the negative and positive feedback regulatory loops that a priori exist in nature, and the combination of which leads to system instability (Robertson, 1991; Seaborg, 1999). This property of feedback regulatory loops has long been noted and was demonstrated in models of biological systems at various levels of their organization (Mackey, Glass, 1977; Decroly, Goldbeter, 1982; Martinez de la Fuente, 1996; Goldbeter et al., 2001; Harish, Hansel, 2015; Likhoshvai et al., 2015, 2016; Kogai et al., 2017; Khlebodarova et al., 2017, 2018). However, it turned out that this is not the only mechanism that can cause instability in a nonlinear dynamic system.

## Periodicity and discontinuity in the history of life viewed through the prism of a mathematical model

No one doubts today that models of mathematical physics are a powerful tool for understanding the deepest laws of the Universe. Methods of mathematical modeling do not yet play such a role in the science of living systems. However, living systems are part of dynamic systems. They are open and non-linear at all levels of their organization, so the method of mathematical modeling is potentially able to help identify the laws of their functioning. And, the more global the system is, the more fundamental and, at the same time, simple in essence, but not in content, should be the laws that determine system’s functioning.

To develop the idea of the internal causes of the discontinuity in evolution, we studied the evolution of large ecosystems using methods of mathematical modeling. We define large ecosystem as a group of organisms (population) of one species, which we designated as “transit” species. In our models, such population mimics the biota of an ecosystem large enough to be correlated with terrestrial or marine biota. These are traditional logistic models of a frame type, in which the efficiency of reproduction and mortality of organisms depends on population density. According to A.V. Markov, hypothesis that the dynamics of the Phanerozoic marine biota calculated by traditional methods (without amendments) adequately reflects real changes in biodiversity has not been unproved and remains the most convenient and reliable basis for meaningful biological interpretations (Markov, Korotaev, 2007, p. 4).

Evolution is described in models as process of ecosystem self-development (population of a “transit” type), during which there is a local increase in the adaptability of its individuals to the conditions of existence due to mutational variability and natural selection.

Analysis of the dynamics of functioning of such models have showed that living systems with different reproduction methods implement different evolutionary laws of selfdevelopment: “asexual” ecosystems showed stasis, whereas “sexual” ecosystems evolved cyclically (Likhoshvai, Khlebodarova, 2016; Likhoshvai et al., 2017). That is, it turned out that if natural selection in a population is directed towards increasing the adaptability of its individuals to the conditions of existence, then, at a certain stage of its evolution (the occurrence of sexual reproduction), such selection can act as destabilizing factor.

**Fig. 2.**
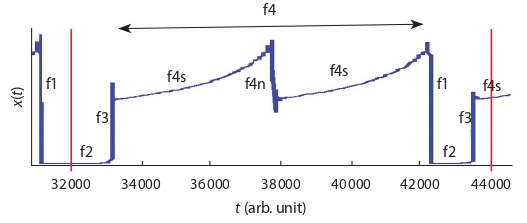
Phases of the x(t) parameter evolution (biota density) demonstrated on the example of one complete oscillation of the x(t) value. Boundaries of the analyzed period are marked with red vertical lines. Phase f1 corresponds to extinction; phase f2 corresponds to the stage of biota development after global extinction; phase f3 corresponds to the stage of explosive growth of biota biodiversity; phase f4 corresponds to the stage of biota development when a high diversity of life forms and a relatively low growth rate are observed. Within the f4 phase, the stasis stage f4s and the local extinction stage f4n are observed.

Moreover, it turned out that these same factors can explain the peculiarities of punctuated evolution observed in the fossil record, such as mass extinctions and phases of rapid diversity increase, as well as phases of stasis diversity, the causes of which are still not understood (Voje, 2016; Voje et al., 2018). Figure 2 shows evolutionary phases of the density parameter of a “transit” population using one full cycle of the parameter value fluctuation. In the model, phases of decrease and increase in the parameter value repeat an unlimited number of times with approximately the same time interval. The exact duration of each phase cannot be predicted, since the oscillatory dynamics observed in the model is chaotic.

One full evolutionary cycle of a “transit” population is completed over time interval t [32,000, 44,000] conv. units, which in the model is ~12,000 conv. units of time (Fig. 2). The concept of fractal evolution (Dieckmann, Law, 1996), which is based on the similarity of laws that regulate the dynamics of population density, variety of species, genera and higher levels of organization of living systems at different time scales, allows to transfer these data when changing the time scale according with a level of organization of living systems with more than a single population. It is easy to verify that if one conv. unit of time equals 50 years, then duration of one evolutionary cycle is close to the species lifespan estimate, and if it equals 500 years, we receive an estimate of the genus lifetime, the durations of which are ~0.5 and ~5.9 million years, respectively, according to (Gingerich, 1976; Severtsov, 1990, 2014). These rough estimates do not prove anything, but suggest that time scales characteristic of dynamic processes at the level of large ecosystems are one order of magnitude larger, that is, such time scales range up to tens of millions of years and cyclic changes in the diversity of Phanerozoic marine biota with a period of 62–63 million years may represent their reflection (Rohde, Muller, 2005; Lieberman, Melott, 2007, 2012; Melott, 2008; Melott, Bambach, 2014, 2017).

Thus, the modeling results have shown that if the efficiency of reproduction and mortality in a population depends on its density and the most adapted individuals, the genetic diversity of which is a result of genome replication errors during self-reproduction, are being selected, then these conditions are sufficient for the formation of cyclical intermittent dynamics of biodiversity in a living system with sexual type of reproduction.

The question arises – what is the origin of cyclicality and intermittency observed in the evolution of life on Earth?

## Global extinctions in the evolutionary history of life on Earth as a reflection of the bistability phenomenon: the hypothesis of two “trees of life”

The idea that the phenomenon of punctuated evolution can be based on the bistability in biological systems has been expressed by V.A. Likhoshvai long ago in the work dedicated to the modeling of the evolution of a simple self-developing living system. It was expressed as an idea of a latent phenotype in a self-developing living system, which represents an internal resource of its evolutionary development (Likhoshvai, Matushkin, 2000, 2004). Subsequently, when applied to global ecosystems, this idea was transformed into the hypothesis of the two “trees of life”.

Here it should be noted that Ch. Darwin defined the diversity of living things on Earth as the “tree of life”. Such comparison very accurately reflects the deepest essence of life, which constantly gives rise to new thin branches of species during its continuous evolutionary development that can eventually form into new genera, types, classes, etc., but can also dry out and disappear (Darwin, 1991).

**Fig. 3.**
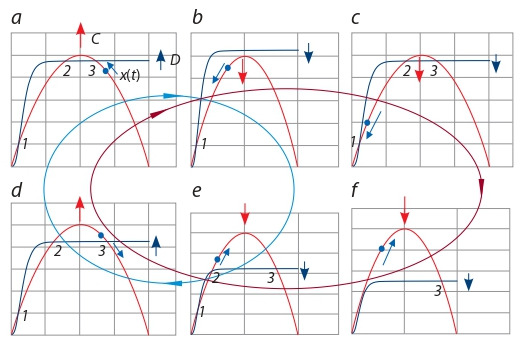
Charts of the reproduction C (red curve) and mortality D (blue curve) functions at different time moments of system evolution. 1 – stable stationary state xmin ; 2 – unstable stationary state xmdl ; 3 – stable stationary state xmax ; blue dot – current х(t) value; colored arrows indicate the direction of evolution (change) of the parameters; blue oval corresponds to the contour of local extinctions and red oval – to global ones.

The most common characteristics of the “tree of life” are biota density and species diversity. These characteristics are reflected in our model as the population density of a “transit” species, which at each moment in time depends on the ratio between the rates of self-reproduction and mortality of its individuals. Analysis of the behaviour of functions that dedescribe changes in these parameters at different time moments depending on the density of a “transit” population have shown that evolving ecosystems with asexual type of reproduction have only one stable state, while for ecosystems with sexual type of reproduction the bistability is possible, that is, two stable stationary states, each of which can be interpreted as the “tree of life”, one of which is being manifested and the other is not. Moreover, if evolution is directed towards improving the adaptability of individuals of a “transit” species to habitat conditions, which should be accompanied by niche expansion and increased utilization of resources, then at some point in time the stability of the manifested state becomes lost and the system jumps into a new steady state that existed before, but was unmanifested. The result of such transition can be interpreted as sudden “disappearance” of old species followed by explosive appearance of new types, that is, the change of one “tree of life” to another. From a mathematical point of view, such event is not unusual in dynamic nonlinear systems. Figure 3 demonstrates the mechanism of local and global extinctions depending on the rates of change of functions describing self-reproduction C (red curve) and mortality D (blue curve) of individuals of a “transit” population at different moments of its evolution.

The intersection of functions C and D corresponds to the stationary states of the system, which can be stable (xmin and xmax) or unstable (xmdl). If current value of density and biodiversity of biota х(t) is located near the stable stationary state, it falls into the region of its attraction and will tend to either xmax (see Fig. 3, a, d, e) or xmin (see Fig. 3, c). The fact that at the same time moment there is one more stable stationary state does not affect the state of the system, since x(t) value falls outside the region of its attraction and the system cannot get into it without external influence. Therefore, we can assume that at the time moment described in Fig. 3, c, stationary state xmin is manifested and stationary state xmax is not. In Fig. 3, a, d, e, on the contrary, stationary state xmax is manifested, whereas stationary state xmin is not.

Since the system evolves over time towards biota size and diversity increase, value of the x(t) parameter increases, while the attraction region of the manifested stationary state decreases and approaches the stationary state xmdl, so that at some point they merge and disappear. At this time moment we observe only one stationary state in the system – either xmin (see Fig. 3, b) or xmax (see Fig. 3, f ), which passes from the unmanifested state to the manifested one. Since at this time moment the x(t) value is significantly different from the value of the manifested stationary state (see Fig. 3, b, f ), an explosive change in the x(t) value is observed. We believe that a rapid change in system parameters during the transition from one state to another can be a reflection of the uneven evolutionary rates observed in phylogenetic studies (Nichol et al., 1993; Pagel et al., 2006; Wolf et al., 2006; Palmer et al., 2012).

It also follows from these data that local extinctions (blue outline) are associated with fluctuations in the current density and diversity of biota х(t) in the attraction region of the stable stationary state xmax (see Fig. 3, a, d ), while global extinctions are associated with the loss of stationary state stability and the х(t) transition to the attraction region of the stationary state xmin, which at this time moment becomes single, similar to that shown in Fig. 3, b. It is this transition that we interpret as the change of one “tree of life” to another.

Thus, we came to the conclusion that adaptation of organisms to the habitat conditions as a result of gradual accumulation of mutations (the evolution) may by itself be one of the causes of instability in a living system, which manifested itself as periodically occurring mass extinctions of biota. However, this instability manifested itself only at a certain stage of the evolution of living systems and was associated with the development of sexual dimorphism. This does not contradict with the fact that during certain periods of life on Earth mass extinctions could coincide with planetary environmental disasters or be provoked by them.

## Conclusion

Analysis of the causes of global extinctions in the Earth’s history have shown that, although abiogenic factors are recognized as prevailing and their various combinations can explain most mass extinctions described in the Earth’s fossil record, they do not explain such aspects of the evolutionary process as periodic discontinuity and uneven evolution of living organisms. However, these are evolutionary characteristics that are manifested at all known levels of organization of living systems – from molecular level to biosphere as a whole. It has now been proven that “spasmodicity” of evolution at the paleontological level is reflected on the molecular level (Nichol et al., 1993; Pagel et al., 2006; Wolf et al., 2006; Palmer et al., 2012).

We believe that in addition to external factors, there are other, internal, reasons for the occurrence of global extinctions of terrestrial biota. According to our hypothesis, these internal factors are associated with the phenomenon of bistability, which occurs only in ecosystems with prevalent sexual reproduction. The fossil record of life on Earth over the past 500 million years reflects the life history of just such organisms. Our hypothesis suggests that even with no global catastrophes of an abiotic nature, extinctions in the evolution of living organisms would happen anyway. The possibility of this is evidenced by the existence of extinctions that are not yet associated with global catastrophes of an abiotic nature, as well as the evidence of serious external influences that were not accompanied by extinctions (Archibald et al., 2010).

We believe that the bistability phenomenon should be manifested in the evolution of a living system at all levels of its organization. And at least at the cellular level, we have demonstrated the contribution of bistability phenomenon to the evolution of cellular complexity (Likhoshvai, Khlebodarova, 2017; Khlebodarova, Likhoshvai, 2018, 2019). There is no doubt that at the level of the entire Earth’s biota the bistability phenomenon should interfere with the abiogenic factors observed in the fossil record of life on Earth. This is evidenced by the extinction cycle with a period of ~140 million years, although it was dimly manifested (Rohde, Muller, 2005), which can be associated with the frequency of glaciations preceding extinctions (Veizer et al., 2000); as well as by the extinction cycle with a period close to 26–27 million years, which was manifested during the last 250 million years (Raup, Sepkoski, 1984, 1986; Sepkoski, 1989; Roberts, Mannion, 2019) and coincided with the dating of volcano craters (Rampino, Caldeira, 2015).

As for the rather strict cyclicity of marine extinctions manifested over the last 500 million years, the period of which was ~63 million years (Rohde, Muller, 2005; Lieberman, Melott, 2007, 2012; Melott, 2008; Melott, Bambach, 2014, 2017), both the empirical data on the ~60 million-years periodicity of the Sr87/Sr86 ratio change in marine sediments (Melott et al., 2012), which indicates the possibility of cyclicity associated with motion of tectonic plates on Earth, as well as modeling data on the fluctuations of the Sun’s large-scale magnetic field with the periodicity of 66 million years (Baker, Flood, 2015), did not conclusively link them to the periodicity of global extinctions.

At this stage, the modeling results do not explain the existence of such periodicity of extinctions. For this, the model is too simple. The dynamics of changes in the biota density observed in the model makes it possible to rather roughly reproduce, with a change in the time scale, the oscillation period characteristic of the specific level of organization of living systems. However, these estimates suggest that the time scales characteristic of dynamic processes at the level of large ecosystems or even the entire Earth, are tens of millions of years. At the moment, this question remains open.

## Conflict of interest

The authors declare no conflict of interest.
